# Integration of digital health applications into the German healthcare system: development of “The DiGA-Care Path”

**DOI:** 10.3389/frhs.2024.1372522

**Published:** 2024-03-13

**Authors:** G. D. Giebel, C. Abels, K. Börchers, B. Kampka, S. Neusser, H. R. Cissarek, F. Plescher, J. Wasem, N. Blase

**Affiliations:** ^1^ Institute for Healthcare Management and Research, University of Duisburg-Essen, Essen, Germany; ^2^ QM BÖRCHERS CONSULTING+, Herne, Germany

**Keywords:** digital health application, DHA, Digitale Gesundheitsanwendungen, DiGA, care path, mHealth, mobile app

## Abstract

**Introduction:**

Since 2019 people who have insured in the German statutory health insurance are entitled to use certified apps called the Digitale Gesundheitsanwendungen [Digital Health Applications (DiGAs)]. The prerequisite for this is that an app certified as DiGA and suitable for their diagnosis exists. The DiGA can then either be prescribed by a physician or psychotherapist or requested by the patient from the statutory health insurance fund. Given the novelty of this type of healthcare, the implementation of a DiGA should be closely monitored to identify potential weaknesses and achieve quality improvements. To enable an analysis of the supply of DiGAs step-by-step, we aimed to create the DiGA-Care Path.

**Methods:**

We conducted three steps to create the DiGA-Care Path. First, a knowledge base was created based on a structured literature research matched with knowledge gathered from the superordinate research project “QuaSiApps” funded by the German Federal Joint Committee. Second, we aimed to create an “ideal-typical” DiGA-Care Path using a flowchart. Third, based on the first path, a final path was developed using the graphical modeling language “Event-Driven Process Chain.”

**Results:**

The DiGA-Care Path was developed to depict the supply of DiGAs in Germany. The final path is constituted by a “main path” as well as a corresponding “sub-path”. While the “main path” focuses more on the supply environment in which a DiGA is used, the “sub-path” depicts the supply delivered by the DiGA itself. Besides the process itself, the paths include relevant actors to indicate responsibilities for individual process steps.

**Discussion:**

The DiGA-Care Path helps to analyze the current supply of DiGAs step-by-step. Thereby, each step can be investigated in detail to identify problems and to detect further steps where quality improvements can be enabled. Depending on the perspective, focused either on the supply environment, or the supply delivered by the DiGA itself, the “main path” or the “sub-path” can be used, respectively. Besides the potential of the DiGA-Care Path to improve the current supply of DiGAs, it can help as an orientation for international policymakers or further stakeholders either to develop their own integration of apps into healthcare systems or for international manufacturers to consider entering the German market.

## 1 Introduction

Since the introduction of the Apple iPhone in 2007, mHealth apps are on the rise and permit multiple patient benefits. These benefits can be achieved in five domains: educational health applications, applications to contact healthcare professionals, applications to check the personal health records, personal care applications, and social networking applications (1).

To help patients benefit from opportunities through mHealth apps, the German Bundestag passed the Digital Care Act (DVG) in 2019. This act established specific mobile as well as web apps also known as Digitale Gesundheitsanwendungen [Digital Health Applications (DiGAs)] as an integral part of the German healthcare system (2). Other European countries such as France and Austria are also considering establishing similar concepts (3). In addition, efforts are being made at the European level to establish a harmonized authorization (4).

DiGAs are not limited to mobile apps; they can also exist in the form of web applications or include other devices [such as virtual reality (VR) glasses]. To become a DiGA, an app has to go through a testing procedure, the “Fast-Track Process for Digital Health Applications (DiGA) according to Section 139e SGB V” (5, 6). The basic requirements for a DiGA tested in this process are as follows: (1) The app must be a European conformity (CE)-certified medical product (risk class I or IIa); (2) the main function of the app is based on digital technologies; (3) the app supports the recognition, monitoring, treatment, or alleviation of diseases or the recognition, treatment, alleviation, or compensation of injuries or disabilities [§33a Social Code Book V (SGB V)]. An approval for apps focused on primary prevention is not provided. After passing the Fast-Track Process, the app will be registered in the DiGA directory managed by the Federal Institute for Drugs and Medical Devices (BfArM) and is reimbursable by the German statutory health insurance.

In general, there are two categories of listing in the directory: (1) DiGA with a provisional listing or (2) DiGA with a final listing. The decision depends on the availability of a comparative study that is suitable to prove a positive healthcare effect. If such a proof exists, a DiGA can directly be final listed in the directory. If manufacturers cannot present a suitable study, they can apply for provisional listing. Nevertheless, all listed DiGA must fulfill requirements such as security, functional capability, quality, data protection, and information security, regardless of their listing status (provisional or final). A corresponding study to prove the positive healthcare effect can be carried out retrospectively as part of a trial phase lasting up to 1 year. If there is a prospect of evidence, a decision may be made at the request of the manufacturer to grant a maximum of a further 12 months for the proof (5, 6).

There are two valid types of positive healthcare effects: Either in the form of a medical benefit or in the form of a patient-relevant improvement of structure and processes (§139e Abs. 2 SGB V). Prior to the approval study, a target patient group has to be determined according to the International Classification of Diseases (ICD-10). Reimbursement for a DiGA is only provided for this designated patient group (5, 6).

The DiGA prices are regulated within §134 SGB V. They differ between the first year and subsequent years. In the first year, the manufacturer is free to determine a price. However, fixed reference price groups must be taken into account depending on the indication and the category of positive healthcare effect. From the 13th month, prices apply that are negotiated between the manufacturer and the National Association of Statutory Health Insurance Funds (GKV-Spitzenverband). If no agreement is reached, an independent arbitration board decides on the price.

Once listed, the manufacturer still has some obligations. In case of significant changes to the DiGA, the BfArM has to be informed. Furthermore, the manufacturer must ensure that information in the DiGA directory, in the distribution platform, or on the application website, is up-to-date and complete. With regard to further requirements, continuous maintenance, reassessment, and further development of the technical and organizational data protection and information security measures must be ensured. This also includes penetration tests. Further requirements are listed within the guide for manufacturers, service providers, and users provided by the BfArM (5, 6) or the Digital Health Applications Ordinance (DiGAV) (7).

Currently, the DiGA directory includes 56 DiGAs. Thereof, 24 DiGAs are provisionally listed and 32 DiGAs are final listed. Six DiGAs could not prove their positive healthcare effect and were subsequently delisted. DiGAs are approved for a broad range of diseases, such as mental illnesses, cardiovascular diseases, cancer, diseases of the muscles, bones, and joints, or hormonal or metabolic diseases (8). Overall, the demand for DiGAs is increasing. While the health insurance funds reimbursed just under 40,000 DiGAs in the first year (October 202 –September 2021), the number rose to over 200,000 reimbursements last year (October 2022–September 2023) (3).

Although safety and clinical performance are tested in the context of the medical device approval process [*Medical Device Regulation (MDR)*, Annex I Chapter 1] and further test criteria such as data protection, information security, interoperability, and further quality requirements are part of the Fast-Track test procedure (5, 6), several problems can emerge within the use of a DiGA. These were found in 10 different categories: (1) validity, (2) usability, (3) technology, (4) use and adherence, (5) data privacy and security, (6) patient–physician relationship, (7) knowledge and skills, (8) individuality, (9) implementation, and (10) costs. On a more abstract level, these are problems concerned either with the DiGAs themselves or their integration into the healthcare system (9).

To address these existing problems and to ensure a high quality of DiGAs, we established the QuaSiApps project with the aim to develop a comprehensive, continuous quality assurance system for DiGAs (10, 11). The project is funded by the German Federal Joint Committee (12).

According to the International Organization for Standardization (ISO) 9000 standard, quality is “the degree to which a set of inherent characteristics fulfills requirements” (13). Thus, a fundamental need to determine the quality of a DiGA and its supply is to know the requirements and gain clarity about the exact procedures and the respective tasks of the concerned parties. One way to guarantee such clarity is the visualization in the form of a care path as the ideal-typical path for the defined patient groups with its decisive diagnostic services in chronological order (14).

Care paths are a methodological basis for the development of quality assurance procedures. Despite some indifferent results, it is now well documented that care paths—or their implementation as a control and standardization instrument for processes—are effective and useful in the context of quality management and quality assurance (15, 16). However, the requirements for the creation of care paths are complex, especially because the fundamental question of care paths: “Who does what with whom at what time” (17) is complex and challenging with regard to the various interfaces (including service providers, payers, and patients). We searched for a clear definition, and a precise depiction, of the ideal-typical care path in the context of DiGAs to build on our quality assurance system. Since our research on guidelines or care paths for DiGA did not result in any eligible results, we determined to create the actual DiGA-Care Path by ourselves. In addition to the benefits that the DiGA-Care Path brings to our project, it can also help national and international stakeholders and interested parties to understand the German DiGA-Care System and look at it in detail.

Hence, the aim of this research was twofold. First, we collected available evidence on the supply of DiGAs in Germany to create a clear understanding of the process, and second, we developed the DiGA-Care Path upon this base.

## 2 Materials and methods

We pursued a three-stage process to develop the DiGA-Care Path. First, we conducted a structured literature research to identify relevant articles and legal standards describing the supply of DiGAs. Therefore, we searched in scientific databases such as MEDLINE via PubMed and Embase for websites of DiGA-relevant stakeholders in the German healthcare system as well as relevant laws and acts in the context of healthcare supply and especially DiGAs. Hereby, the aim was not to conduct an exhaustive research identifying all the relevant literature in the context of these apps but to gain a broad and reliable knowledge base on which the DiGA-Care Path can be developed further.

Second, a first version of the DiGA-Care Path (see Supplementary Figure S1) was developed. It summarizes the ideal-typical path of DiGA patient groups with decisive services in the chronological sequence. In addition, the actors involved and the DiGA interventions (e.g., the performance of a DiGA application anamnesis) are depicted.

This first care path is based on various sources of knowledge that were generated both within the QuaSiApps project and with the help of external literature. A flowchart was modeled based on: (1) statements of patients (18) and experts from a qualitative survey within the project; (2) a case-based problem outline of medical ethical implications in the use of a DiGA (19); (3) the four medical ethical principles of autonomy, care, non-harm, and justice (20); and (4) the model professional code for doctors working in Germany (21).

Based on this draft, we discussed and decided that the model would benefit from a modeling language that enables a more complex branching than “yes/no-decisions.” We also wanted to add the relevant application software (in our context, the DiGA) and the relevant organizational unit (e.g., patient, DiGA manufacturer, or service provider) to the respective process steps in the path. Since the Event-Driven Process Chain (EPC) modeling language developed at the Institute for Information Systems (IWi) at the University of Saarland, Germany (22), offers these possibilities, we used it.

To depict the processes, the EPC uses alternating events and functions that are connected through arrows, showing what is called the “Control Flow.” In case a function can be followed by different “Events,” “Connectors” are used to branch the “Control Flow.” In our case we used “XOR (Either-or)” and “OR (And-or) Connectors.”

**Figure 1:**
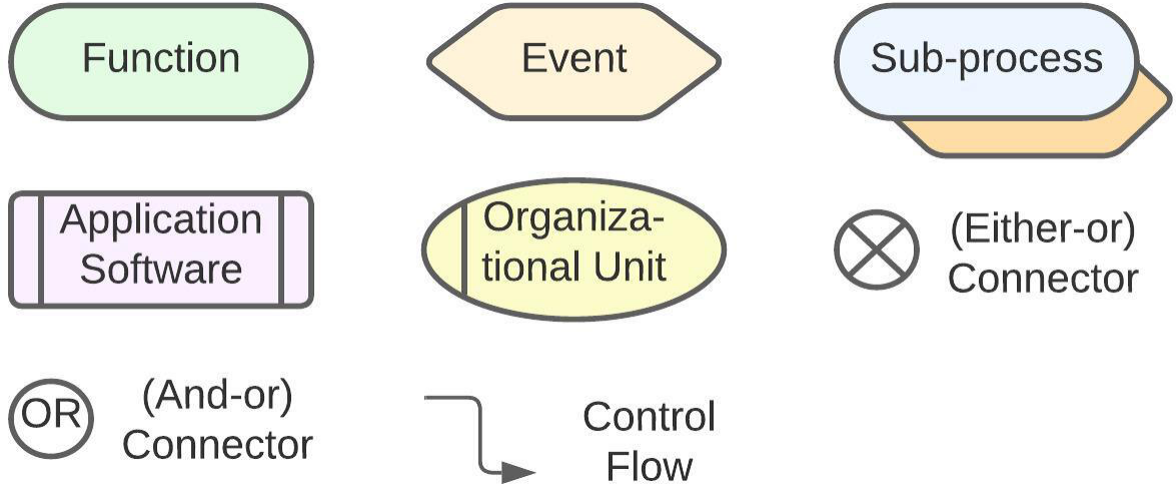
Basic components of the event-driven process chain.

To clarify which executing body is responsible for a function, we modeled the respective “Organizational Unit” to each function. Where indicated, we added the DiGA as an “Application Software.” To indicate the conjunction between a main- and a sub-path, an interface was implemented by a “Sub-process” (cf. Figure 1).

To implement the DiGA-Care Path, we used Lucidchart (23), a web-based diagramming application from Lucid Software Inc. that allows visualization of charts and diagrams, organizational structures, and especially processes.

Finally, after developing the DiGA-Care Path, we sent it out to independent DiGA-experts without conflicts of interest with the research project and asked them after 1 week within an online meeting to evaluate whether the path is correct and intuitive to understand.

## 3 Results

To develop the DiGA-Care Path, we started with the structured literature search yielding four legal standards (cf. Table 1), six articles (9, 19, 24–27), the QuaSiApps project accompanying working paper (10), a book about DiGAs (28, 29) as well as the Fast-Track Process for DiGA (5, 6) (cf. Table 2).

**Table 1:** Legal standards relevant in the context of DiGA.

Reference	Description
§33a SGB V	Statutory anchoring of DiGA contains the legal definition and regulates the entitlement of persons with statutory health insurance to the use of DiGA.
	According to the paragraph, DiGA are defined as low-risk class (I or IIa) medical devices whose primary function relies substantially on digital technologies and which are intended to assist in the detection, monitoring, treatment, or mitigation of disease or the detection, treatment, mitigation, or compensation of injury or disability in the insured or in the care provided by healthcare practitioners.
	Furthermore, it includes the procurement channels either through a healthcare practitioner or the authorization by the health insurance. A fundamental requirement for DiGA use irrespective of the procurement channel is a medical indication.
§134 SGB V	Regulation about the remuneration for DiGA use.
§139e SGB V	Regulates the way in which manufacturers can apply for a listing of their medical apps in the DiGA-directory. The basic requirement is a proof of evidence in three areas: (1) the app meets the requirements for safety, functional capability, and quality, including the interoperability of the medical device; (2) the app meets the requirements for data protection and ensures data security in accordance with the state of the art; and (3) the app has a positive healthcare effect.
DiGAV	Determines how the Digital Care Act (DVG), which has incorporated DiGA into the SGB V, is to be implemented. The DiGAV describes in more detail than the DVG how manufacturers can prove that they or their products meet the legal requirements. For example, the ordinance contains specific checklists that manufacturers must use to verify that IT security requirements are met. In contrast to the law, the ordinance also regulates the costs, the procedural sequence, and the precise contents of the DiGA-directory (30).

**Table 2:** Sources included from the structured research that were used to build the DiGA-Care Path.

No.	Author(s)/law	Description	Language
1	Kuhn et al. (19)	A case-based problem outline of the medical–ethical implications of DiGA use.	German
2	Giebel et al. (9)	A review of problems and barriers that have and might have an impact on the supply of DiGAs.	English
3	Börchers and Kampka (10)	A working paper including a detailed description of the QuaSiApps project, FAQs in the context of the project as well as a comprehensive project glossary.	German
4	Schelling (27)	A handout of the association of Statutory Health Insurance Physicians, Bavaria, with advice and recommendation for the avoidance of liability in the context of DiGA.	German
5	BfArM (5, 6)	A Guide for Manufacturers, Service Providers, and Users for the Fast-Track Process for DiGAs.	English/German
7	Sauermann et al. (26)	An article describing basics in the context of DiGAs.	English
8	Geier (24)	An article that describes the status quo in 2021 and recent as well as prospective challenges.	German
9	Brönneke et al. (28, 29)	A comprehensive book about the integration of DiGAs into the German healthcare system.	English/German
10	Haserück and Lau (25)	A short article about the integration of DiGAs into the healthcare supply and the viewpoint of physicians.	German

Based on the included articles and the knowledge generated within the QuaSiApps project, authors KB and BK developed a first DiGA-Care Path (September 2022) as a basis for discussion. After a project meeting that served for a discussion on the consortium of gathered ideas, corrections and improvements were highlighted and we consented to rework the path and implement it in more detail.

Mainly the need for an either–or branching after an activity or function led to the decision to choose another more comprehensive modeling language. Thus, we chose the EPC allowing this modeling.

**Figure 2:**
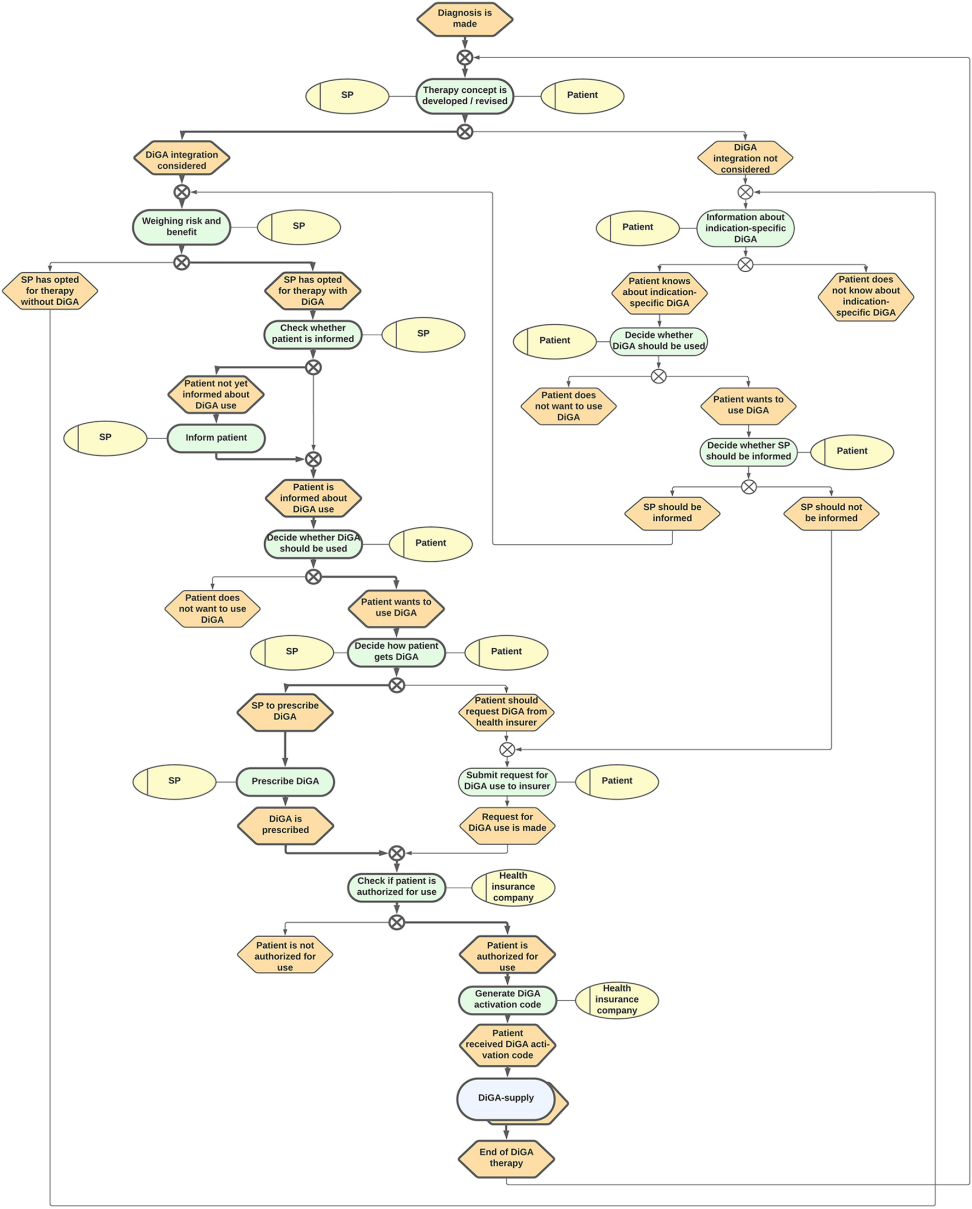
DiGA-Care Path 1: supply environment in which DiGAs are used. SP, Service provider (either physician or psychotherapist); DiGA, Digitale Gesundheitsanwendung (Digital Health Application).

**Figure 3:**
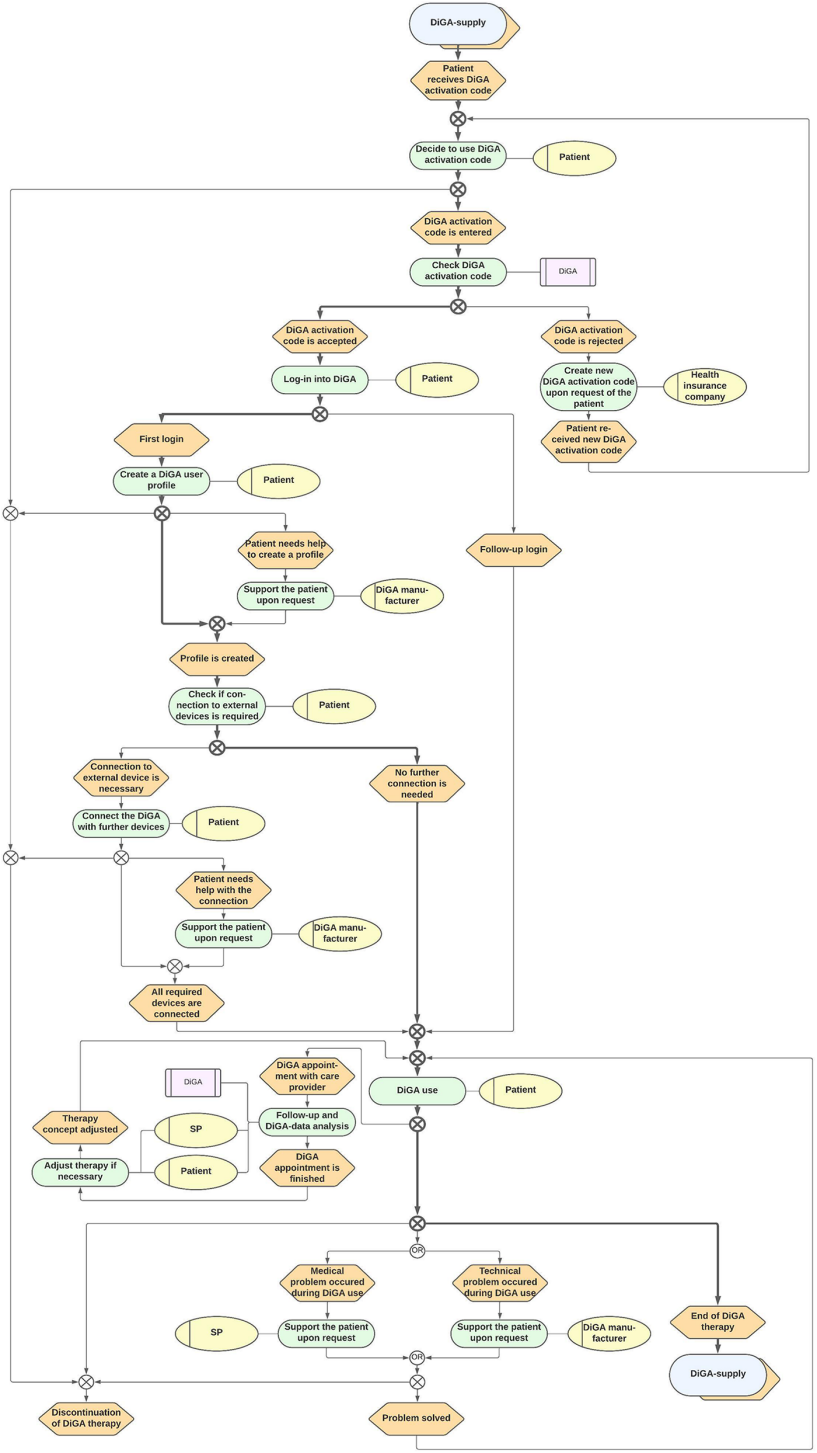
DiGA-Care Path 2: supply by the DiGA itself. SP, Service provider (either physician or psychotherapist); DiGA, Digitale Gesundheitsanwendung (Digital Health Application).

To reduce the complexity, we decided to separate the DiGA-Care Path into a main path as well as a sub-path contained therein. The main path represents the supply environment in which the DiGA is used (cf. Figure 2). The sub-path includes the supply by the DiGA itself (cf. Figure 3). According to the consensus, the final paths were iteratively modeled with feedback loops including the whole consortium.

Once the paths were developed, we sent them to four independent experts for review. We then held an online meeting together with the experts to discuss the accuracy and comprehensibility of the DiGA-Care Paths. Both paths were judged to be easily understandable as well as correct in content. Nevertheless, it was suggested to emphasize the most frequently followed path within the DiGA-Care Path. To find this, we used the DiGA-Report 2022 from Germany's largest health insurance company. According to the report, 85% of users receive their DiGA via a prescription and 15% receive it via a direct request to their health insurance company (31).

The final paths are represented in Figures 2, 3. Since they were judged to be self-explanatory, only a short, written description of the process is given here.

### 3.1 Supply environment in which DiGAs are used

The “Supply environment in which DiGAs are used” is depicted in care path 1 (cf. Figure 2). Care path 1 is the higher-level process that includes the “Supply by the DiGA itself” (cf. Figure 3) as a sub-process.

The starting point of DiGA supply is always the diagnosis of a disease made by a healthcare practitioner for which a DiGA exists. After the diagnosis, the healthcare practitioner and patient together develop a concept of therapy that can either include a DiGA or not. If the healthcare practitioner estimates the risk–benefit assessment positive, he offers to inform the patient about the use of the DiGA. If the patient is already experienced with the appropriate DiGA, he can waive the medical information. If the patient declines the use of the DiGA at any point, the app does not become part of the healthcare supply.

If the patient and healthcare practitioner both decide to integrate DiGA into the therapy, the usual path will continue with a prescription made by the healthcare practitioner. Subsequently, the health insurance company of the patient will check if the patient is entitled to use the DiGA or not. If their review results in a positive assessment, the patient receives an activation code to use the DiGA.

Otherwise, if either the healthcare practitioner is not aware of the DiGA or declines its usage within the concept of therapy, the patient himself can request for the DiGA when given the suitable diagnosis (and no relevant contraindications) directly from his healthcare insurance company.

Irrespective of the way of procurement, the patient always has the option to ask or inform the healthcare practitioner about the DiGA use.

### 3.2 Supply by the DiGA itself

The supply provided by the DiGA itself is shown in care path 2 (Figure 3). This path is a sub-process of the supply environment process. After the patient receives his activation code, he decides whether to use it or not. If he does not use it, the DiGA therapy is canceled. Otherwise, the code is checked by the DiGA automatically. Once the code is accepted and the user creates a profile and connects any additional devices, the patient can use the DiGA. In case of any appointments due to the use, the healthcare practitioner and patient analyze the DiGA data and possibly adjust the DiGA therapy concept. If there are any medical or technical problems during use, the patient should get support from his healthcare practitioner or the manufacturer, respectively. If the problem is solved, the patient can continue the use. At the end of the “DiGA utilization period,” the patient is redirected to the care path 1 and should evaluate (alone or together with the healthcare practitioner) whether a further use is indicated or not.

## 4 Discussion

### 4.1 Principal findings

The DiGA-Care Path was developed to depict the supply of DiGAs in the German healthcare system. It enables researchers, policymakers, and further stakeholders to analyze the supply of a DiGA step-by-step, to identify the parties involved in each case and to locate potential weaknesses, problems, and quality indicators at individual points.

In principle, it can be assumed that the German healthcare system improves through the integration of DiGAs. This assumption is mainly based on two reasons: (1) A DiGA must prove either a medical benefit or a patient-relevant improvement of structure and processes (§139e Abs. 2 SGB V), and (2) the positive attitude of outpatient-care general practitioners, physicians, and psychotherapists (32) as well as physical therapists, occupational therapists, and speech-language pathologists (33) toward DiGAs. Nevertheless, there are several problems and barriers that might impede the prescription and use of a DiGA (9, 32). To face those challenges as well as to optimize the integration and maximize the subsequent positive effects of DiGAs, different approaches should be pursued. One is the implementation of quality assurance. The starting point for quality assurance should be a clear understanding of the care process. Therefore, we gathered and analyzed laws, literature, and project knowledge to build on the DiGA-Care Path.

Even if the high quality of the DiGA itself and its optimal integration into existing care are factors that affect patient benefit, other factors such as the actual use of the app must be taken into account. To verify the actual patient benefit, a new law provides the implementation of application-related performance measurements. This law, the “Act to accelerate the digitalization of the healthcare system” [Digital-Gesetz (DigiG), not yet in force] (34) will also bring further changes such as a closer integration of DiGAs into existing processes and the extension of the risk class of DiGAs to class IIb according to the MDR.

Even if the developed path correctly represents the theoretical requirements for the supply, at some points in the supply reality, the supply of DiGAs could deviate from the path. Such an example we know from focus groups with patients (18) within the QuaSiApps project: In some cases, younger, tech-savvy family members take on the role of DiGA manufacturers and support users with technical questions.

Especially, the closer integration into existing processes is welcomed by many stakeholders because concerns were raised that DiGAs should not be used without integration into the standard care provided by physicians and psychotherapists [e.g., the German Psychotherapists Association (35), the German Diabetes Society (36), the Professional Association for Orthopaedics and Trauma Surgery e.V (37), or Heidel et al. (38)]. Nevertheless, insufficient reimbursement of medical services in the context of DiGAs might be a problem or barrier toward the prescription of DiGAs by physicians or psychotherapists (9, 32, 38). Such changes should always be kept in mind when using the DiGA-Care Path. From time to time, it should be reflected if the DiGA-care is changing and if subsequent changes should be implemented in the DiGA-Care Path.

Furthermore, even if not regulated yet, other stakeholders such as physical therapists, occupational therapists, and speech-language pathologists might play a role in the supply of DiGAs. Thus, for example, a survey with 150 therapists found that 87.3% indicated a positive intention to use a DiGA. In addition, it was to be expected that patients would use DiGAs incorrectly and that errors could therefore occur during training with an app. Therefore, it is necessary to examine to what extent a DiGA can replace an in-person therapy, or whether supplementary or partial replacement use is preferable (33).

Even if apps are becoming more ubiquitous in healthcare systems all over the world, most countries, however, did not or only rudimentarily regulate their integration and use. Also, the European Union only dictates that apps must fulfill the criteria of the MDR to become medical products and to receive admission to healthcare systems and in the secondary healthcare market. Further regulations about how apps should be integrated into care do not exist on this level. Germany took it one step further and created a legal basis for the integration of distinct apps (DiGAs).

But even if there is a legal basis, the integration of DiGAs into care paths is uncertain. Therefore, the National Association of the Statutory Health Insurance Funds emphasized: “DiGA must be integrated into the care paths. To this end, the potential for digitalization in treatment and networking across service sectors must be exploited” (39). While we depicted the general DiGA-Care Path that is independent of indication, the integration of DiGAs should also be considered in the context of disease-specific care paths, especially by medical professional societies.

### 4.2 Limitations

DiGA-care in Germany is a very complex system. Nevertheless, we aimed to illustrate it as clearly organized as possible. Therefore, we had to neglect some aspects. Such an aspect is especially the contact between the patient and the healthcare practitioner. Even if we depicted this contact at some points of our path, it should be emphasized that the patient should always have the option to contact the respective healthcare practitioner at any time during the DiGA-Care Path.

We decided to use the EPC to represent the German DiGA supply. Even if the EPC was originally developed to model business processes (22), it proved to be the right modeling language for our purpose. This was mainly because it provides the possibility to use Either–Or Connectors, which were necessary to detail the complex system, and because of the easy readability. Since there is a variety of other modeling languages (e.g., the BPMN), translating the DiGA-Care Path into other forms of representation could also be considered.

The DiGA-Care Path was reviewed by four independent DiGA-experts. It can therefore be assumed that in principle it correctly maps the supply of DiGAs. Nevertheless, the review did not include a broad range of different stakeholders. Further stakeholders, such as patients, physicians, manufacturers, or policymakers, should also be asked about their assessment of the appropriateness of the path. Hence, the path should be subject to further discussion and could therefore be subject to change in the future.

A further limitation was the non-systematic evidence collection we used to develop the DiGA-Care Path. We did not conduct a systematic literature review or a systematic survey of experts. Therefore, there is a risk that the evidence is incomplete. Nevertheless, we are convinced that based on the knowledge gathered during the project, together with the literature and the review by the four experts, our DiGA-Care Path adequately represents the DiGA-care.

## 5 Conclusions

We analyzed and subsequently visualized the DiGA-Care Path using the graphical modeling language EPC. Thereby, the DiGA-care process becomes transparent and can be further investigated in detail. The developed DiGA-Care Path serves as a solid foundation to examine the weaknesses of the current situation as well as to indicate areas where one can start to improve care. Furthermore, it provides an overview of the German DiGA supply. Thus, the DiGA-Care Path can either be used as an inspiration for policymakers or further stakeholders to develop their own integration of mHealth apps into healthcare systems, or for international manufacturers to consider entering the German market.

## Data Availability

The original contributions presented in the study are included in the article/Supplementary Material, further inquiries can be directed to the corresponding author.
