# Corrigendum: Integration of digital health applications into the German healthcare system: development of “The DiGA-Care Path”

**DOI:** 10.3389/frhs.2024.1416456

**Published:** 2024-04-26

**Authors:** G. D. Giebel, C. Abels, K. Börchers, B. Kampka, S. Neusser, H. R. Cissarek, F. Plescher, J. Wasem, N. Blase

**Affiliations:** ^1^ Institute for Healthcare Management and Research, University of Duisburg-Essen, Essen, Germany; ^2^ QM BÖRCHERS CONSULTING+, Herne, Germany

**Keywords:** digital health application, DHA, Digitale Gesundheitsanwendungen, DiGA, care path, mHealth, mobile app

A Corrigendum on Integration of digital health applications into the German healthcare system: development of “The DiGA-Care Path” By Giebel GD, Abels C, Börchers K, Kampka B, Neusser S, Cissarek HR, Plescher F, Wasem J, Blase N (2024). Front Health Serv. 4:1372522. doi: 10.3389/frhs.2024.1372522

In the published article, there was an error in Supplementary Figure S1. A preliminary, German-language version of the attachment was uploaded by mistake. The correct and final version of Supplementary Figure S1 appears below.


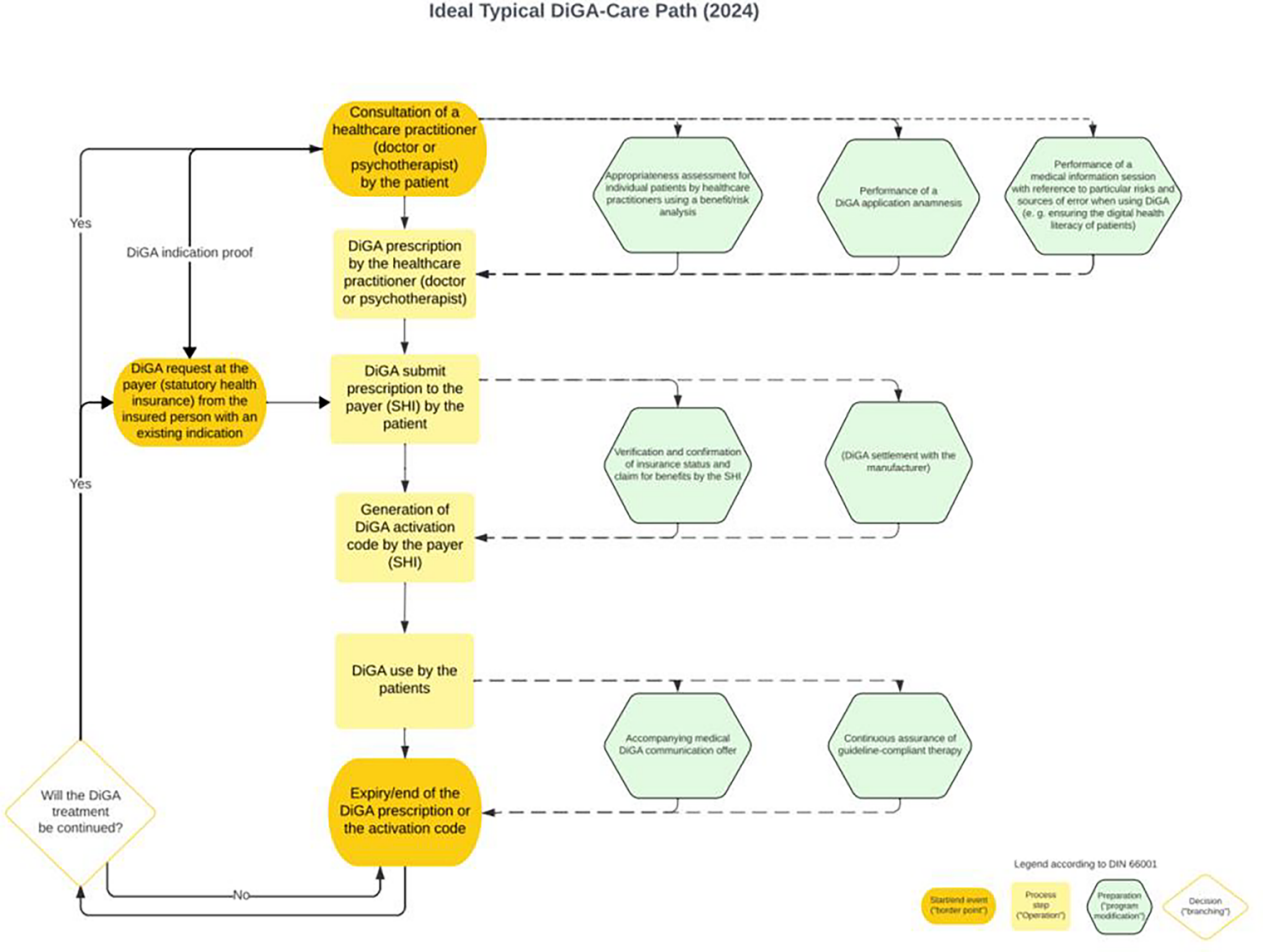


The authors apologize for this error and state that this does not change the scientific conclusions of the article in any way. The original article has been updated.

