# Supplementary figures and images for: Integration of digital health applications into the German healthcare system: development of “The DiGA-Care Path”
Source: Front Health Serv. 2024 Mar 13;4:1372522. doi: 10.3389/frhs.2024.1372522 (PMC10966120; doi:10.3389/frhs.2024.1372522)

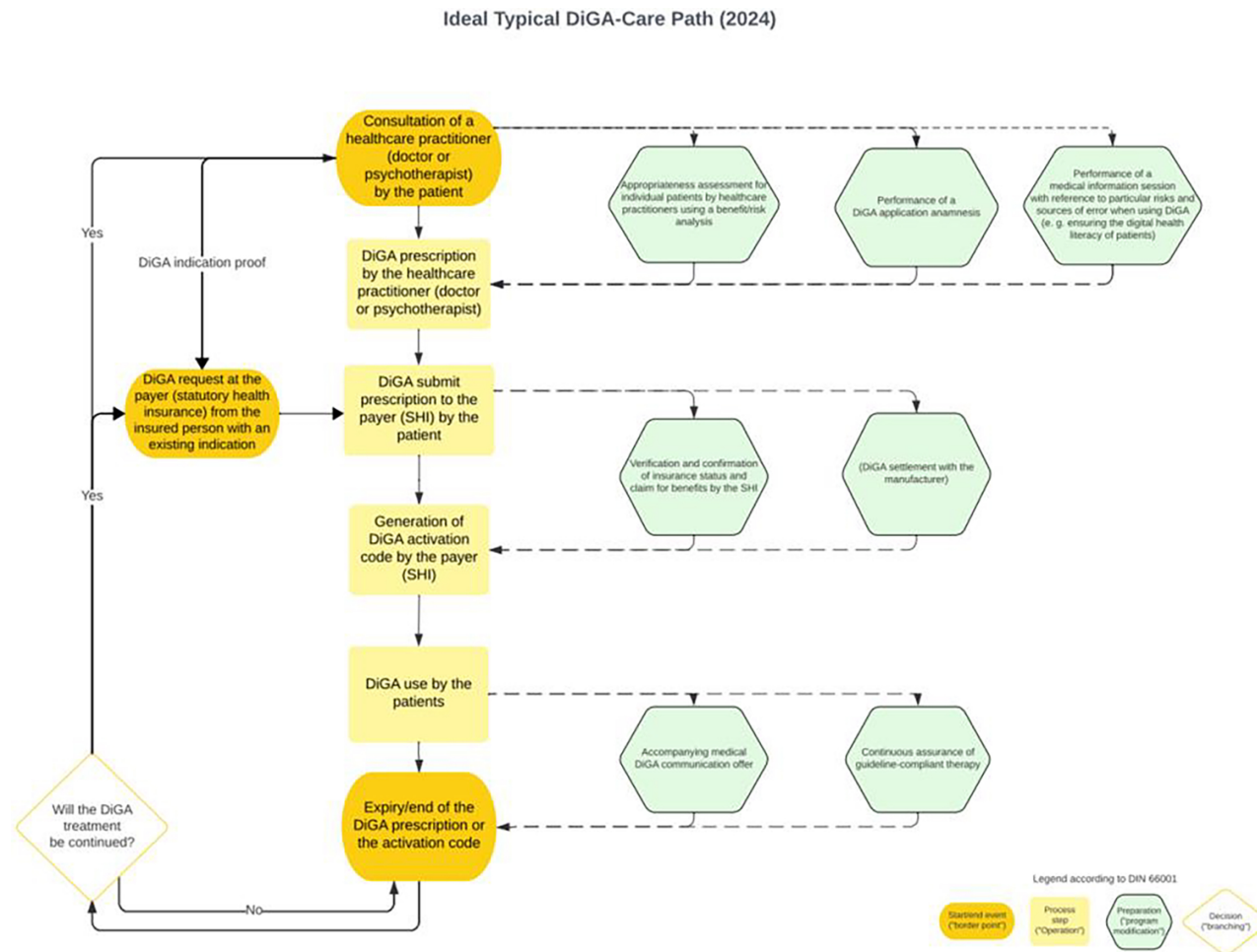

**Supplementary Figure 1.** First version of the ideal-typical DiGA-Care path.

Supplement: Supplementary file 1 [file Image1.pdf]
